# Temporal trends in anxiety and depression prevalence and their association with adverse outcomes in patients hospitalized for acute exacerbations of chronic obstructive pulmonary disease in Beijing, China, from 2004 to 2020

**DOI:** 10.3389/fpsyt.2022.996451

**Published:** 2022-10-31

**Authors:** Lin Feng, Jiachen Li, Xiaoshuang Lv, Shuilian Chu, Changwei Li, Ruiyuan Zhang, Xi Cao, Lirong Liang

**Affiliations:** ^1^Department of Clinical Epidemiology, Beijing Institute of Respiratory Medicine and Beijing Chao-Yang Hospital, Capital Medical University, Beijing, China; ^2^Department of Respiratory and Critical Care Medicine, Beijing Institute of Respiratory Medicine and Beijing Chao-Yang Hospital, Capital Medical University, Beijing, China; ^3^Department of Epidemiology, Tulane University School of Public Health and Tropical Medicine, New Orleans, LA, United States

**Keywords:** anxiety, depression, chronic obstructive pulmonary disease, temporal trend, influence, China

## Abstract

**Aims:**

To investigate the temporal trend in anxiety and/or depression prevalence in patients hospitalized for acute exacerbation of chronic obstructive pulmonary disease (AECOPD) in Beijing and their association with adverse outcomes.

**Materials and methods:**

Hospital admission records from 2004 to 2020 with a primary discharge diagnosis of AECOPD were retrieved from Beijing Public Health Information Centre database. The anxiety and depression were identified from discharge diagnoses of each record. Joinpoint regression was used to analyze the temporal trend and calculate the annual percentage change (APC) for the prevalence of anxiety and/or depression. Generalized linear model was used to analyze the associations between anxiety and/or depression and patients’ adverse outcomes.

**Results:**

A total of 382,125 records were included, most of which were male (66.0%) and aged ≥ 75 years (59.7%). Three segments in the temporal trend were observed, with a mild increase during 2004–2009 (APC: 5.9%, 95% CI: -14.9 to 31.7%), followed by a sharply increase during 2009–2012 (APC: 60.4%, 95% CI: 10.6 to 132.7%), then stabilized at about 3% during 2012–2020 (APC: 1.9%, 95% CI: -0.4 to 4.3%). On average, anxiety, and/or depression was more prevalent in females, the aged and those admitted in secondary hospitals (all *P* < 0.001). Patients with anxiety and/or depression had lower in-hospital mortality (IHM) (OR = 0.74, 95% CI: 0.63–0.88), but longer hospital stay (OR = 1.10, 95% CI: 1.07–1.13), more medical costs (OR = 1.12, 95% CI: 1.08–1.17) and higher risks of readmission for AECOPD at 30-, 90-, 180-day, and 1-year (ORs ranged from 1.22 to 1.51).

**Conclusion:**

The prevalence of anxiety and/or depression in patients hospitalized for AECOPD in Beijing stabilized at approximately 3% after 2012. Anxiety and/or depression is associated with a heavier burden on patients, health care, and medical insurance systems. Appropriate diagnosis and effective treatment of anxiety and depression is crucial for patients with AECOPD.

## Introduction

Chronic Obstructive Pulmonary Disease (COPD) is one of the top three global causes of death and top ten global causes of disability-adjusted life years (DALYs) (1). Anxiety and depression are common in patients with COPD, with an estimated prevalence of 6–74% and 8–80%, respectively (2–7). As important comorbidities in COPD, anxiety and depression are associated with increased risks of exacerbations (2, 8), emergency care use (9), and medical costs (10), contributing to a substantial disease burden of COPD (6).

It’s worth noting that most of results reported in previous studies related to the prevalence, associated factors and influences of depression and/or anxiety were restricted to stable COPD patients from outpatient or community settings (2, 9, 11). Different from those with stable COPD, patients hospitalized for acute exacerbation of chronic obstructive pulmonary disease (AECOPD) are often in a severer physical condition and worse quality of life (12), which are closely related to the occurrence of depression and/or anxiety symptoms (3). Meanwhile the presence of depression and/or anxiety could also worsen the condition of COPD as well as the quality of life of patients (13–15). Accordingly, the anxiety and depression could be more prevalent among patients hospitalized for AECOPD than those with stable COPD, but limited data has been reported in the previous study.

Apart from the point prevalence, the temporal trends of anxiety and depression prevalence in AECOPD are also of paramount importance in understanding the changes in mental health status over time, which has significant implications for health policy and healthcare provision in systematically influencing clinical practice (16). Although of high priority (12, 17), the existing literature fails to assess the temporal trends in the prevalence of anxiety and depression among those hospitalized for AECOPD.

In the meantime, there is still controversy regarding the impact of anxiety and depression on the prognosis of patients hospitalized for AECOPD. Some authors have reported a worse disease progression (18–20), while others have failed to find any link between anxiety nor depression and a worse prognosis (8, 21). More evidence from large-sample representative study is needed.

Therefore, the present study focused on patients hospitalized for AECOPD with two aims: (1) to describe the temporal trends of anxiety and/or depression prevalence among patients hospitalized for AECOPD in Beijing; (2) to estimate the associations between comorbid anxiety and/or depression and the in-hospital outcomes as well as the risks of readmission for AECOPD within 1-year after discharge.

## Materials and methods

### Study design

This study is a city-wide electronic medical records (EMR) -based study of patients hospitalized for AECOPD in Beijing, with a cross-sectional study design to describe the temporal trends of the prevalence of anxiety and/or depression, along with a retrospective cohort study design to estimate the associations between anxiety and/or depression and the in-hospital outcomes as well as the risks of readmission for AECOPD at 30-, 90-, 180-days, and 1-year.

The present study was approved by the Research Ethics Board of Beijing Chaoyang Hospital (2018-ke-303). Data were de-identified before analysis. It is impossible to identify patients at the individual level either in this article or in the retrieved database. Given the anonymous and mandatory nature of the data, informed consent was not required or necessary.

### Data source

AECOPD hospitalization records were retrieved form a hospital discharge database operated by the Beijing Public Health Information Centre. This database covers discharge records from all secondary- and tertiary-level hospitals in Beijing. As only secondary- and tertiary-level hospitals could provide inpatient service, this database provides good representative of hospitalized patients in Beijing and can be used to analyze hospitalization outcomes. These patient-level records contain data on patient’s demographic characteristics, hospital name, date of admission, discharge diagnoses along with corresponding International Classification of Diseases, 10th Revision (ICD-10) codes, and so on. All hospitalization records for patients aged ≥ 20 years with a primary discharge diagnosis of AECOPD (ICD-10 codes of J44.0–J44.9) from January 1st 2004 to December 30th 2020 were included in the current analyses.

### Measurement

Anxiety (ICD-10 codes of F40.0-F40.9, F41.1-F41.9) and depression (ICD-10 codes of F32.0-F32.9, F33.0-F33.9, F41.2) were identified from discharge diagnoses in each hospitalization record. Patients with only anxiety diagnosis, only depression diagnosis, both anxiety and depression diagnoses were considered as in the only anxiety subtype, only depression subtype, and both anxiety and depression subtype. The other combined diseases were also identified from discharge diagnoses and the corresponding Charlson Comorbidity Index (CCI) was calculated (22). Use of mechanical ventilation (MV), including non-invasive MV and invasive MV, was determined using ICD-10 code J15.501, which was available in the dataset from 2012 to 2020. The length of hospital stay (LOHS) was defined as days from admission to discharge of each record. The medical cost was total cost during each hospitalization and was converted into 2020 Chinese Yuan (CNY) values (1 CNY = 0.145 US dollar) based on the year-specific health care consumer price index of China (23). The readmission for acute exacerbation was defined as another hospitalization with a primary diagnosis of AECOPD (ICD-10 codes of J44.0–J44.9) happened afterward (within 30-, 90-, 180-days, 1-year), which was identified in our hospital discharge database.

### Statistical analysis

Descriptive statistics were presented as means and standard deviations or medians and interquartile ranges for continuous variables with or without normal distributions and as frequencies and percentages for categorical variables. Characteristics between groups were compared using χ^2^-test or Fisher’s exact test for nominal categorical data, the Mantel-Haenszel Chi-Square test for ordered categorical data, and the Wilcoxon rank sum test or Kruskal-Wallis test for continuous variables without normal distribution, respectively.

The prevalence of anxiety and/or depression and the subtypes (only depression, only anxiety, both anxiety and depression) were presented annually in the total hospitalization records for AECOPD as well as the subgroups defined by gender, age group and institute level. The temporal trend analyses were conducted using Joinpoint Regression Program developed by United States National Cancer Institute. The turning points in the temporal trend of the prevalence of anxiety and/or depression could be identified and the annual percentage change (APC) for each time segment could be calculated. The program assumes that proportions changed at a constant percentage every year on a log scale in each time segment. The average annual percent change (AAPC)—a weighted average of APCs from the Joinpoint models, with weights equal to the length of the APC interval—was also computed as a summary measure of the trend over the whole observation period.

The generalized linear models were used to investigate the associations between co-diagnosis of anxiety and/or depression and patients’ prognoses, with a random effect to account for multiple hospitalization records of one patient. The binominal distribution and logit link were used to investigate the associations between co-diagnosis of anxiety and/or depression and receiving MV, in-hospital mortality (IHM) and readmission for AECOPD after discharge. The gamma distribution and log link were used to investigate the associations between co-diagnosis of anxiety and/or depression and LOHS, medical costs. Model covariates included admission year, gender, age, CCI, and institute level. Statistical analyses were performed using SAS 9.4 (SAS Institute Inc., Cary, NC, USA). Statistical significance was set as two-sided *P* < 0.05.

## Results

### Patients’ characteristics and the prevalence of anxiety and/or depression

A total of 382,125 patient discharge records were identified, submitted by 78 tertiary hospitals and 77 secondary hospitals. Most patients (59.6%) were 75 years old or older and 66.0% of them were male. The types of anxiety and depression diagnosis and the corresponding ICD-10 code were shown in Supplementary Table 1. As shown in Table 1, a total of 2.1% (7,912 of 382,125) patients had a co-diagnosis of anxiety and/or depression. The prevalence of anxiety and/or depression was higher for females than for males and increased with age, with the highest prevalence seen among patients aged ≥ 75 years (all *P* < 0.001). Details about the prevalence of its subtypes are showed in Supplementary Table 2.

**TABLE 1:** The characteristics and the prevalence of anxiety and/or depression among patients hospitalized for AECOPD from 2004 to 2020 in Beijing, China.

	Total	With anxiety and/or depression	*P*
Overall	382,125	7,912 (2.1%)	
**Gender**			
Male	252,055	4,438 (1.8%)	<0.001
Female	130,070	3,474 (2.7%)	
**Age**			
Median (IQR)	77.0 (69.0–83.0)	79.0 (72.0–84.0)	
20–59 years	28,893	367 (1.3%)	<0.001
60–74 years	125,190	2,145 (1.7%)	
≥ 75 years	228,042	5,400 (2.4%)	
P trend			<0.001
**Institute level**			
Secondary hospitals	108,861	2,424 (2.2%)	<0.001
Tertiary hospitals	269,824	5,368 (2.0%)	
**Charlson comorbidity index**			
0	148,424	3,182 (2.1%)	0.012
1	128,378	2,656 (2.1%)	
≥ 2	105,157	2,074 (2.0%)	
P trend			0.003

IQR, interquartile range; Institute level missing (*n* = 3,440), Charlson Comorbidity Index missing (*n* = 166).

### Trends of anxiety and/or depression in patients hospitalized for acute exacerbation of chronic obstructive pulmonary disease 2004–2020

**FIGURE 1:**
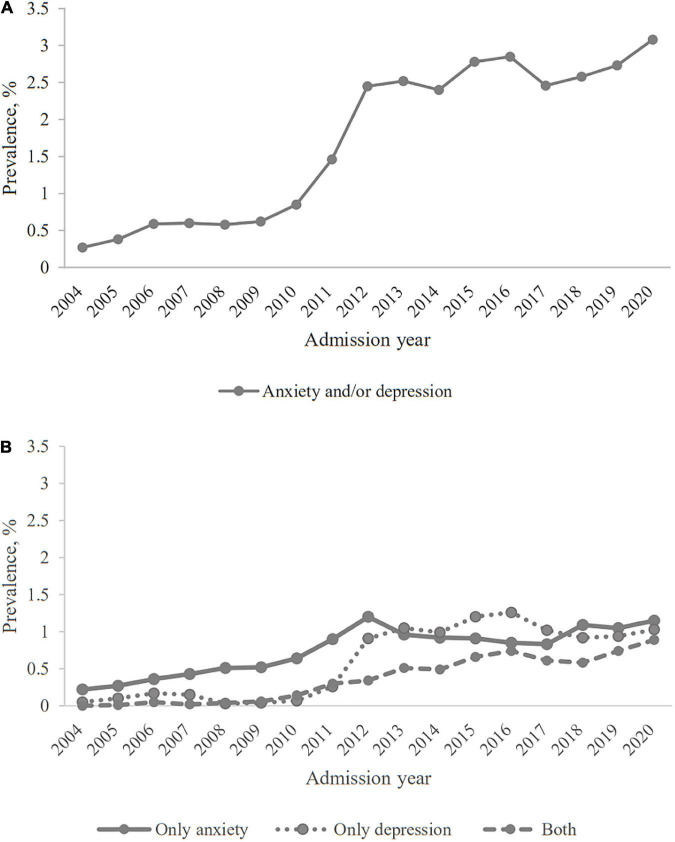
The prevalence of anxiety and/or depression among patients hospitalized for AECOPD in Beijing. **(A)** Anxiety and/or depression; **(B)** three subtypes of anxiety and/or depression.

Figure 1A shows the prevalence of anxiety and/or depression and its subtypes among patients with AECOPD for each calendar year. During the observation period, the prevalence increased significantly from 0.3% in 2004 to 3.1% in 2020. Trends in the prevalence of three subtypes exhibit different patterns (Figure 1B). During 2004–2012, the prevalence of only anxiety increased continuously, and then stabilized at about 1%. The prevalence of only depression and the prevalence of both anxiety and depression stayed at about 0.1% during 2004–2011. In 2012, the former increased sharply and stabilized at about 1%, while the latter has been increasing since 2011 and reaching about 1% in 2020. The annual prevalence data are in Supplementary Table 3.

**FIGURE 2:**
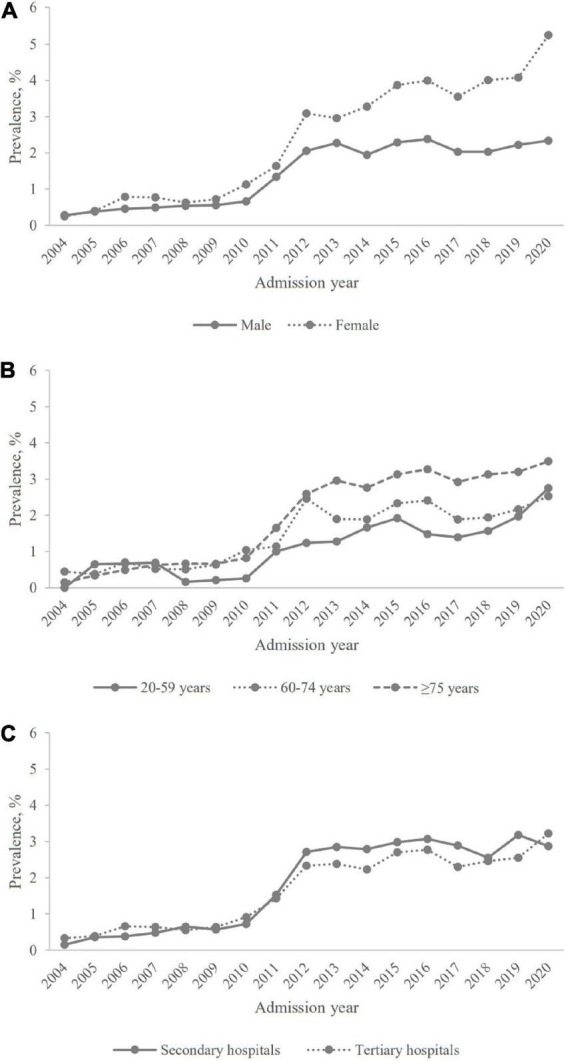
The prevalence of anxiety and/or depression among patients hospitalized for AECOPD in Beijing, stratified by gender, age group, and institute level. **(A)** Gender; **(B)** age group; **(C)** institute level.

The temporal trends in the prevalence of anxiety and/or depression were compared by subgroups and the corresponding results are displayed in Figure 2. The prevalence for female patients were close to those for male patients from 2004 to 2011, and then became almost twice as those for male patients. The prevalence for three age groups all fluctuated during study period, and mainly manifested as the older age the higher the prevalence. However, the prevalence for patients aged 20–59 years old exceeded that for patients aged 60–74 years old in 2020. The temporal trends in the prevalence for patients in secondary hospitals and for those in tertiary hospitals were similar during study period, with sharp increases in 2011 and 2012. The temporal trends in the prevalence of three subtypes stratified by subgroups are showed in Supplementary Figures 1–3.

In Joinpoint Regression, the prevalence of anxiety and/or depression increased significantly during study period with an AAPC of 12.3% [95% confidence interval (CI): 3.1 to 22.3%]. Two turning points with three segments were observed in the temporal trend of prevalence, with a mild increase during 2004–2009 (APC: 5.9%, 95% CI: -14.9 to 31.7%), followed by a sharply increase during 2009–2012 (APC: 60.4%, 95% CI: 10.6 to 132.7%), and then stabilized during 2012–2020 (APC: 1.9%, 95% CI: -0.4 to 4.3%). In subgroups, APCs for female patients increased during 2012–2020 with 5.8% (95% CI: 2.6 to 9.0%) while male patients did not have a significant change during that period (APC: 0.6%, 95% CI: -1.9 to 3.1%). Over the entire observation period, patients aged 20–59 had a continuous upward trend without a significant turning point; while in 2012, one turning point was found in those aged 60–74; and in 2009 and 2012, two turning points were found in those aged ≥ 75. During 2009–2012, the APC of secondary hospitals was higher than that of tertiary hospitals (73.1% vs. 54.9%) (details in Table 2).

**TABLE 2:** Trend analysis of the prevalence of anxiety and/or depression among patients hospitalized for AECOPD by gender, age group and institute level from 2004 to 2020.

	Overall trend		Trend segment 1^a^			Trend segment 2			Trend segment 3		
				
	AAPC (95% CI)	*P*	Period	APC (95% CI)	*P*	Period	APC (95% CI)	*P*	Period	APC (95% CI)	*P*
Overall	12.3 (3.1, 22.3)	0.008	2004–2009	5.9 (–14.9, 31.7)	0.570	2009–2012	60.4 (10.6, 132.7)	0.018	2012–2020	1.9 (–0.4, 4.3)	0.093
**Gender**											
Male	11.6 (1.6, 22.6)	0.023	2004–2009	6.1 (–15.9, 33.8)	0.577	2009–2012	60.1 (4.4, 145.6)	0.035	2012–2020	0.6 (–1.9, 3.1)	0.605
Female	14.1 (2.3, 27.3)	0.018	2004–2009	4.6 (–22.0, 40.2)	0.738	2009–2012	61.7 (2.5, 155.3)	0.041	2012–2020	5.8 (2.6, 9.0)	0.002
**Age group**											
20–59	10.1 (4.9, 15.6)	0.001	2005–2020^b^	10.1 (4.9, 15.6)	<0.001						
60–74	14.7 (9.4, 20.2)	< 0.001	2004–2012	29.6 (17.6, 42.9)	<0.001	2012–2020	1.4 (–2.1, 5.1)	0.399			
≥ 75	14.1 (5.7, 23.1)	0.001	2004–2009	8.7 (–11.9, 34.2)	0.391	2009–2012	63.5 (20.6,121.7)	0.005	2012–2020	2.7 (0.8, 4.6)	0.009
**Institute level**											
Secondary	14.5 (5.4, 24.3)	0.001	2004–2009	9.1 (–12.2, 35.6)	0.387	2009–2012	73.1 (21.9,145.7)	0.006	2012–2020	1.0 (–1.0, 3.1)	0.281
Tertiary	11.5 (–0.6,25.0)	0.062	2004–2009	4.9 (–21.6, 40.4)	0.717	2009–2012	54.9 (–6.2,155.8)	0.080	2012–2020	2.3 (–0.9, 5.7)	0.143

AAPC, average annual percentage change; APC, annual percentage change; CI, confidence interval.

^a^Trend segment identified by Joinpoint regression.

^b^No patient aged 20–59 year diagnosed with depression and/or anxiety in 2004.

### The associations of anxiety and/or depression and patients’ outcomes

As shown in Table 3, patients with anxiety and/or depression had lower IHM (2.1% vs. 3.0%), longer LOHS (13.0 days vs. 12.5 days), more medical cost (19,515 CNY vs. 17,004 CNY), but similar mechanical ventilation rates (5.5% vs. 5.2%, *P* = 0.356). Among those alive at discharge and followed up at least for 1 year, patients with anxiety and/or depression had higher risks of readmission for AECOPD at 30-day (20.5% vs. 16.9%), 90-day (32.95 vs. 25.7%), 180-day (42.2% vs. 33.2%), and 1-year (53.6% vs. 43.3%) (all *P* < 0.001). Patients in three different subtypes had different mechanical ventilation rates, LOHS and risks of readmission for AECOPD after discharge (all *P* < 0.001).

**TABLE 3:** The differences in in-hospital outcomes and the risks of readmission for AECOPD after discharge between those with anxiety and/or depression and those without.

	With anxiety and/or depression			Subtypes			
		
	Yes	NO	*P*	Only depression	Only anxiety	Both	*P*
	*N* = 7,912	*N* = 37,4213		*N* = 2,911	*N* = 3,288	*N* = 1,713	
Receiving mechanical ventilation, *n* (%)^a^	384 (5.5)	13,678 (5.2)	0.356	146 (5.3)	180 (6.8)	58 (3.7)	<0.001
In-hospital mortality, *n* (%)^b^	162 (2.1)	10,708 (3.0)	<0.001	55 (1.9)	77 (2.4)	30 (1.8)	0.265
Length of hospital stay, day, median (IQR)	13.0 (9.0–18.0)	12.5 (8.9–17.2)	<0.001	12.9 (9.0–17.0)	13.0 (9.0–19.8)	13.0 (9.0–17.0)	<0.001
Medical cost, CNY^c^, median (IQR)	19,515 (13,127–30,160)	17,004 (11,445–25,808)	<0.001	19,148 (12,978–28,924)	19,653 (13,046–31,224)	19,783 (13,700–30,407)	0.064
30–day readmission for AECOPD, *n* (%)^d^	1,493 (20.5)	5,8970 (16.9)	<0.001	625 (21.9)	597 (18.6)	364 (21.6)	0.003
90-day readmission for AECOPD, *n* (%)^d^	2,404 (32.9)	8,9939 (25.7)	<0.001	954 (33.4)	993 (30.9)	601 (35.7)	0.002
180-day readmission for AECOPD, *n* (%)^d^	3,080 (42.2)	11,6129 (33.2)	<0.001	1,225 (42.9)	1,260 (39.2)	768 (45.65)	<0.001
1-year readmission for AECOPD, *n* (%)^d^	3,910 (53.6)	15,1169 (43.3)	<0.001	1,511 (52.9)	1,636 (51.0)	949 (56.4)	0.001

IQR, interquartile range; Fisher’s exact test for binary variables; Wilcoxon rank sum test and Kruskal-Wallis test for continuous variables.

^a^Hospitalization recodes in 2012–2020, *N* = 268,456.

^b^Hospitalization recodes in 2007–2020, *N* = 361,776.

^c^Converted to CNY 2020.

^d^Hospitalization recodes alive at discharge in 2004–2019, *N* = 356,862.

After multivariate adjustment, patients with anxiety and/or depression still had a lower risk of IHM (OR = 0.74, 95% CI: 0.63 to 0.88), but longer LOHS (OR = 1.10, 95% CI: 1.07 to 1.13), more medical costs (OR = 1.12, 95% CI: 1.08 to 1.17) and higher risks of readmission for AECOPD after discharge at 30-day (OR = 1.22, 95% CI: 1.04 to 1.43), 90-day (OR = 1.39, 95% CI: 1.24 to 1.55), 180-day (OR = 1.44, 95% CI: 1.31 to 1.59) and 1-year (OR = 1.51, 95% CI: 1.38 to 1.64). Details in Table 4.

**TABLE 4:** The multivariate analyses of the associations between depression or anxiety and patients’ in-hospital outcomes and the risks of readmission for AECOPD after discharge.

	Model 1		Model 2	
		
	Odds ratio (95% CI)	*P*	Odds ratio (95% CI)	*P*
Receiving mechanical ventilation^a^	1.06 (0.74, 1.51)	0.759	1.09 (0.76, 1.56)	0.648
In-hospital mortality^b^	0.73 (0.62, 0.86)	<0.001	0.74 (0.63, 0.88)	<0.001
Length of hospital stay	1.09 (1.05, 1.12)	<0.001	1.10 (1.07, 1.13)	<0.001
Medical cost	1.10 (1.05, 1.15)	<0.001	1.12 (1.08, 1.17)	<0.001
30-day readmission for AECOPD^c^	1.11 (0.94, 1.30)	0.214	1.22 (1.04, 1.43)	0.015
90-day readmission for AECOPD^c^	1.28 (1.14, 1.43)	<0.001	1.39 (1.24, 1.55)	<0.001
180-day readmission for AECOPD^c^	1.35 (1.22, 1.48)	<0.001	1.44 (1.31, 1.59)	<0.001
1-year readmission for AECOPD^c^	1.43 (1.31, 1.56)	<0.001	1.51 (1.38, 1.64)	<0.001

Model 1: adjusted for admission year.

Model 2: adjusted for sex, continuous age, continuous Charlson Comorbidity Index, institute level and admission year.

CI, confidence interval.

^a^Hospitalization recodes in 2012–2020, *N* = 268,456.

^b^Hospitalization recodes in 2007–2020, *N* = 361,776.

^c^Hospitalization recodes alive at discharge in 2004–2019, *N* = 356,862.

The multivariate analyses of only depression, only anxiety, both depression and anxiety on the in-hospital outcomes and readmission for AECOPD were shown in Supplementary Table 4. No significant difference was observed among three different subtypes except for the LOHS (*P* for interaction, 0.020).

## Discussion

During the observation period of this EMR-based observational study, the prevalence of anxiety and/or depression increased sharply from 2009 to 2012, before stabilizing at approximately 3%. The prevalence of subtypes of anxiety and/or depression and subgroups stratified by gender, age group and institute level all increased, however, they showed varying temporal patterns. Anxiety and/or depression was more prevalent among the elderly, female patients and those hospitalized in secondary hospitals. Patients with anxiety and/or depression had a lower risk of IHM, but longer LOHS and higher medical costs, as well as higher risks of readmission for AECOPD after discharge within 1 year.

During 2009–2012, the prevalence of anxiety and/or depression among AECOPD inpatients increased sharply. An improvement of the regional mental health service system leading by local government could be responsible for this increase (24). All general hospitals in Beijing were required to establish psychiatric departments (25). Psychiatric specialists provided extensive training to clinicians in general hospitals, and marked increases in psychiatric beds and psychiatric professionals were observed (25). Our results indicate that the systemic improvement in the diagnostic capacity of common mental disorders in general hospitals, particularly in secondary hospitals. Since 2012, the prevalence of depression and/or anxiety among AECOPD inpatients has remained stable, fluctuating between 2.4 and 3.1%. This may be indicative of the true prevalence of comorbid anxiety and depression among AECOPD inpatients.

Diagnosis of anxiety and depression is essential to delivering effective psychiatric treatments for AECOPD patients. In recent years, approximately 3% AECOPD inpatients were co-diagnosed with anxiety and/or depression in Beijing, which is lower than those reported in Taiwan (depression: 9.0%) and the United States (anxiety: 9.6%; depression: 14.2%) (19, 21). This difference can be attributed to several factors. It relates, in part, to the difficulty in identifying and diagnosing anxiety and depression among AECOPD inpatients. Symptoms of these mental disorders are similar to those of AECOPD, including dyspnea, oppression in chest, palpitations, fatigue, sleep disturbances, loss of appetite, reduced physical activity, and hopelessness (4, 26). Therefore, there is a need for more experienced psychiatric professionals as well as a well-established system to improve future diagnosis capabilities. On the other hand, in previous studies, psychological scales such as the Hospital Anxiety and Depression Scale (HADS) and Hamilton Anxiety Rating Scale (HAMA) were used to detect AECOPD patients suffering from anxiety and/or depression (3, 8, 20). Accordingly, these studies reported higher prevalence of anxiety and depression than we did (ranging from 44.4 to 68.2%) (3, 8). It should be noted, however, that anxiety and depression based on scales may be affected by AECOPD symptoms (27), these measures are not comparable to the clinical diagnoses (28).

The results of another EMR-based study of 26,591 veterans with AECOPD admissions in the United States, of whom 97% were male, showed that those with anxiety and/or depression were younger than those without psychiatric comorbidity, who had an average age of 61.9 years and 3.1 comorbid diagnoses on average (18). By contrast, anxiety and/or depression was more prevalent among the elderly in our study. Perhaps this is due to the difference in the study populations, as veterans may be more likely to get anxiety and depression from their prior war experience (29). Moreover, similar to a previous study, female patients with AECOPD were more likely to experience anxiety and depression (18). This could be explained as female patients are more sensitive to respiratory symptoms, experiencing more negative emotions, thus leading to higher risks of anxiety and depression (30). Furthermore, female COPD patients are more likely to face barriers to receiving appropriate treatment (31). Poor female COPD management may also contribute to the progression of the disease, increasing their risk of depression and anxiety. In addition, an increasing prevalence of anxiety and depression in patients with AECOPD aged 20–59 years in recent years was observed. There is still much uncertainty about the causes, but more attentions should be paid to mental health among young and middle-aged people and explore their influences.

After the COVID-19 pandemic outbreak, the prevalence of anxiety and/or depression increased by about 15% (from 2.7 to 3.1%). This slight increase could be due to a number of factors. As a result of the uncertainties and fears of the virus infection, mass lockdowns and economic recession, people have higher psychological distress during the COVID-19 pandemic (32, 33). Furthermore, COPD patients are more likely to experience disorders of mental illness during COVID-19 pandemic because of worse access to necessary medical care for COPD (34, 35). In view of the fact that our study period ended in 2020, more data in subsequent years are required to determine whether the COVID-19 pandemic has a profound impact on the prevalence of anxiety and depression among patients hospitalized for AECOPD.

In a retrospective study of Taiwan’s health insurance database, which included 4,204 first-ever AECOPD patients, 73% were male and the mean age was 75 years, those dying in hospital (7.4%) had less depression than those survived (9.1%) (21). Similarly, we found AECOPD patients with anxiety and/or depression was associated with a lower risk of IHM. This association has not been fully elucidated. According to previous studies, COPD patients suffering from anxiety and/or depression could not objectively evaluate their condition and might experience subjectively worsening lung disease (36, 37). AECOPD inpatients with anxiety and/or depression may overestimate the severity of an exacerbation and, therefore, are not in as severe physical condition as those without anxiety nor depression. Due to the lack of severity measurements, neither our study nor the other study in Taiwan were able to evaluate this hypothesis. For a more conclusive conclusion, a prospective cohort study is required. Moreover, we observed patients with anxiety and/or depression had longer LOS and spent more money in the hospital. This association was also observed in a prospective cohort study that excluded patients who died during their hospitalization (38). The explanation for this association is still unclear. Even so, anxiety and depression are undoubtedly important burden for AECOPD patients, the health care systems and the medical insurance systems.

In our prospective study of 504 patients with stable COPD, anxiety and depression were associated with an increased risk of acute exacerbations in the following year (11). Similarly, our present study demonstrates that comorbid anxiety and/or depression is associated with increased the risks of 30-, 90-, 180-days, and 1-year readmission for AECOPD in patients hospitalized for AECOPD, which has not been reported before. There are several mechanisms that linking anxiety and depression to the prognosis of COPD. Physiologically, anxiety and depression can activate the sympathetic nervous system and the hypothalamic-pituitary-adrenal axis, leading to a weakened immune system and increased vulnerability to respiratory infection and exacerbation of COPD (39, 40). Psychologically, COPD patients with anxiety and/or depression have decreased self-efficacy, resulting in insufficient management of their diseases manifested as poor medication adherence and participation in pulmonary rehabilitation (41–43).

Anxiety and depression are extra-pulmonary treatable traits of COPD patients (17). In current clinical guideline, there is no evidence that anxiety and depression should be treated differently in the presence of COPD (12). Moreover, recent studies showed that some COPD treatments such as mind-body exercise, breathing-based walking, and pulmonary rehabilitation could also improve patients’ anxiety and depression symptoms (44–46). Multidisciplinary disease management for COPD patients with psychological assessment and follow-up by clinical psychologist also showed promising preliminary results (47). Consequently, it is imperative to identify anxiety and depression in COPD patients, especially those with acute exacerbations, in order to provide them with adequate psychological treatment and ultimately improve their prognosis.

### Strengths and limitations

This is, to the best of our knowledge, the first and largest long-term study conducted in a city over a 17-year period, using a representative database of AECOPD hospitalizations. Additionally, we used the Joinpoint Regression Program to identify points where these trends changed and to estimate the APC to uncover underlying causes, such as changes in policy or program implementation at specific points in time. There are also several limitations should be noted. First, as with all studies utilizing medical records, it was impossible for us to determine whether a hospitalization record was for a patient experiencing their first episode of anxiety and/or depression or for a patient experiencing recurrent episodes. The lack of data in our database also prevented us from examining the potential effects of socioeconomic status, smoking, or alcohol consumption, exacerbation severity, and pharmaceutical treatments on comorbid anxiety and/or depression. Second, anxiety and/or depression is likely to be underdiagnosed in our study population, resulting in systematically underestimated prevalence rates in AECOPD inpatients. And the temporal trends in our study mixed the changes of prevalence and improvements in diagnostic capability. Taken together, our temporal trend does not accurately reflect the “true” epidemic trend in anxiety and/or depression in patients hospitalized for AECOPD. However, we provide with useful information for policymakers regarding the prevalence of anxiety and depression among patients hospitalized with AECOPD. Third, no temporal trend analysis for only anxiety, only depression, both anxiety and depression was conducted because their prevalence are too low to fit the Joinpoint regression model. Fourth, there is inevitably survivor bias in a retrospective cohort, which may lead to a null interpretation for anxiety and depression being associated with patients’ prognoses, particularly IHM. Finally, the data are from Beijing, and our findings may not be generalizable to patients with AECOPD in other provinces of China. Future studies using nationwide records are warranted to better reflect the burden of this condition in China as a whole.

## Conclusion

Our study showed that the prevalence of anxiety and/or depression among patients hospitalized for AECOPD in Beijing stabilized at approximately 3% after a sharp increase during 2009–2012. Anxiety and/or depression is associated with a longer LOS, more medical cost, and higher risk of readmission for AECOPD. It is imperative to identify anxiety and depression in AECOPD patients, and to provide them with adequate psychological treatment and ultimately to reduce the disease burden on patients, health care and medical insurance systems.

## Data availability statement

The original contributions presented in the study are included in the article/Supplementary material, further inquiries can be directed to the corresponding author.

## Ethics statement

The studies involving human participants were reviewed and approved by the Research Ethics Board of Beijing Chaoyang Hospital (2018-ke-303). The ethics committee waived the requirement of written informed consent for participation.

## Author contributions

LF and LL designed the analysis. LL acquired the data resource, supervised the work, and edited the final version of the manuscript. LF analyzed the data and wrote the first draft. JL, XL, SC, CL, RZ, and XC provided expertise as well as edited the contents of the manuscript. All authors contributed to the article and approved the submitted version.

## References

[1] World Health Organization. *The Global Health Observatory, Global Health Estimates: Life Expectancy and Leading Causes of Death and Disability.* (2022). Available online at: https://www.who.int/data/gho/data/themes/mortality-and-global-health-estimates (Accessed February 2022).

[2] Lou P Chen P Zhang P Yu J Wang Y Chen N Effects of smoking, depression, and anxiety on mortality in Copd patients: a prospective study. *Respir Care.* (2014) 59:54–61. 10.4187/respcare.02487 23737545

[3] Long J Ouyang Y Duan H Xiang Z Ma H Ju M Multiple factor analysis of depression and/or anxiety in patients with acute exacerbation chronic obstructive pulmonary disease. *Int J Chron Obstruct Pulmon Dis.* (2020) 15:1449–64. 10.2147/copd.S245842 32606653 PMC7310996

[4] Willgoss TG Yohannes AM. Anxiety disorders in patients with Copd: a systematic review. *Respir Care.* (2013) 58:858–66. 10.4187/respcare.01862 22906542

[5] Zhang MW Ho RC Cheung MW Fu E Mak A. Prevalence of depressive symptoms in patients with chronic obstructive pulmonary disease: a systematic review, meta-analysis and meta-regression. *Gen Hosp Psychiatry.* (2011) 33:217–23. 10.1016/j.genhosppsych.2011.03.009 21601717

[6] Zareifopoulos N Bellou A Spiropoulou A Spiropoulos K. Prevalence, contribution to disease burden and management of comorbid depression and anxiety in chronic obstructive pulmonary disease: a narrative review. *COPD.* (2019) 16:406–17. 10.1080/15412555.2019.1679102 31638445

[7] Yohannes AM Willgoss TG Baldwin RC Connolly MJ. Depression and anxiety in chronic heart failure and chronic obstructive pulmonary disease: prevalence, relevance, clinical implications and management principles. *Int J Geriatr Psychiatry.* (2010) 25:1209–21. 10.1002/gps.2463 20033905

[8] Martinez-Gestoso S Garcia-Sanz MT Carreira JM Salgado FJ Calvo-Alvarez U Doval-Oubina L Impact of anxiety and depression on the prognosis of copd exacerbations. *BMC Pulm Med.* (2022) 22:169. 10.1186/s12890-022-01934-y 35488330 PMC9052487

[9] Blakemore A Dickens C Chew-Graham CA Afzal CW Tomenson B Coventry PA Depression predicts emergency care use in people with chronic obstructive pulmonary disease: a large cohort study in primary care. *Int J Chronic obstruct Pulm Dis.* (2019) 14:1343–53. 10.2147/COPD.S179109 31388297 PMC6607976

[10] Pollok J van Agteren JE Esterman AJ Carson-Chahhoud KV. Psychological therapies for the treatment of depression in chronic obstructive pulmonary disease. *Cochrane Database Syst Rev.* (2019) 3:CD012347. 10.1002/14651858.CD012347.pub2 30838649 PMC6400788

[11] Huang J Bian Y Zhao Y Jin Z Liu L Li G. The impact of depression and anxiety on chronic obstructive pulmonary disease acute exacerbations: a prospective cohort study. *J Affect Disord.* (2021) 281:147–52. 10.1016/j.jad.2020.12.030 33333473

[12] Global Initiative for Chronic Obstructive Lung Disease. *Gold Reports 2022.* (2022). Available online at: https://Goldcopd.Org/2022-Gold-Reports-2/ (accessed March 1, 2022).

[13] Salte K Titlestad I Halling A. Depression is associated with poor prognosis in patients with chronic obstructive pulmonary disease - a systematic review. *Dan Med J.* (2015) 62:A5137. 26441395

[14] Pooler A Beech R. Examining the relationship between anxiety and depression and exacerbations of Copd which result in hospital admission: a systematic review. *Int J Chron Obstruct Pulmon Dis.* (2014) 9:315–30. 10.2147/copd.S53255 24729698 PMC3974694

[15] Pedrozo-Pupo JC Campo-Arias A Ceballos-Ospino GA. Quality of life and depression in copd patients in the Colombian Caribbean. *Clin Respir J.* (2021) 15:944–8. 10.1111/crj.13385 33949121

[16] Tran J Norton R Conrad N Rahimian F Canoy D Nazarzadeh M Patterns and temporal trends of comorbidity among adult patients with incident cardiovascular disease in the Uk between 2000 and 2014: a population-based cohort study. *PLoS Med.* (2018) 15:e1002513. 10.1371/journal.pmed.1002513 29509757 PMC5839540

[17] Cardoso J Ferreira AJ Guimarães M Oliveira AS Simão P Sucena M. Treatable traits in copd – a proposed approach. *Int J Chron Obstruct Pulmon Dis.* (2021) 16:3167–82. 10.2147/copd.S330817 34824530 PMC8609199

[18] Abrams TE Vaughan-Sarrazin M Van der Weg MW. Acute exacerbations of chronic obstructive pulmonary disease and the effect of existing psychiatric comorbidity on subsequent mortality. *Psychosomatics.* (2011) 52:441–9. 10.1016/j.psym.2011.03.005 21907063

[19] Singh G Zhang W Kuo YF Sharma G. Association of psychological disorders with 30-day readmission rates in patients with copd. *Chest.* (2016) 149:905–15. 10.1378/chest.15-0449 26204260 PMC4944783

[20] Alqahtani JS Aldabayan YS Aldhahir AM Al Rajeh AM Mandal S Hurst JR. Predictors of 30- and 90-day Copd exacerbation readmission: a prospective cohort study. *Int J Chron Obstruct Pulmon Dis.* (2021) 16:2769–81. 10.2147/COPD.S328030 34675502 PMC8504869

[21] Ho TW Tsai YJ Ruan SY Huang CT Lai F Yu CJ In-hospital and one-year mortality and their predictors in patients hospitalized for first-ever chronic obstructive pulmonary disease exacerbations: a nationwide population-based study. *PLoS One.* (2014) 9:e114866. 10.1371/journal.pone.0114866 25490399 PMC4260959

[22] Charlson ME Pompei P Ales KL MacKenzie CR. A new method of classifying prognostic comorbidity in longitudinal studies: development and validation. *J Chronic Dis.* (1987) 40:373–83. 10.1016/0021-9681(87)90171-8 3558716

[23] Compiled by National Bureau of Statistics China. *China Statistical Yearbook [Internet].* Xicheng: Compiled by National Bureau of Statistics China (2021).

[24] The People’s Government of Beijing Municipality. Guidelines on strengthening the construction and development of Beijing mental health service system. *Bull Beijing Municipal Peoples Govern*. (2011) 28:64–8.

[25] Commission BMH. *Statistical bulletin on the development of health services in beijing*. (2012). Available online at: www.phic.org.cn/ (accessed March 23, 2013).

[26] Underner M Cuvelier A Peiffer G Perriot J Jaafari N. [The influence of anxiety and depression on copd exacerbations]. *Rev Mal Respir.* (2018) 35:604–25. 10.1016/j.rmr.2018.04.004 29937312

[27] Hansen H Beyer N Frolich A Godtfredsen N Bieler T. Inter-day test-retest reproducibility of the Cat, Ccq, Hads and Eq-5d-3l in patients with severe and very severe Copd. *Patient Relat Outcome Meas.* (2021) 12:117–28. 10.2147/PROM.S306352 34104024 PMC8179805

[28] Griffith MF Chen HP Bekelman DB Feemster LC Spece LJ Donovan LM Comorbid anxiety and depression, though underdiagnosed, are not associated with high rates of low-value care in patients with chronic obstructive pulmonary disease. *Ann Am Thorac Soc.* (2021) 18:442–51. 10.1513/AnnalsATS.201912-877OC 33306930 PMC7919148

[29] Inoue C Shawler E Jordan CH Jackson CA. *Veteran and Military Mental Health Issues.* Treasure Island, FL: Statpearls (2022).

[30] Du J Mayer G Hummel S Oetjen N Gronewold N Zafar A Mental health burden in different professions during the final stage of the Covid-19 lockdown in China: cross-sectional survey study. *J Med Internet Res.* (2020) 22:e24240. 10.2196/24240 33197231 PMC7713530

[31] Gut-Gobert C Cavailles A Dixmier A Guillot S Jouneau S Leroyer C Women and Copd: do we need more evidence? *Eur Respir Rev.* (2019) 28:180055. 10.1183/16000617.0055-2018 30814138 PMC9488562

[32] Xiong J Lipsitz O Nasri F Lui LMW Gill H Phan L Impact of covid-19 pandemic on mental health in the general population: a systematic review. *J Affect Disord.* (2020) 277:55–64. 10.1016/j.jad.2020.08.001 32799105 PMC7413844

[33] Shi L Lu ZA Que JY Huang XL Liu L Ran MS Prevalence of and risk factors associated with mental health symptoms among the general population in China during the Coronavirus disease 2019 pandemic. *JAMA Netw Open.* (2020) 3:e2014053. 10.1001/jamanetworkopen.2020.14053 32609353 PMC7330717

[34] Yohannes AM. Copd patients in a Covid-19 society: depression and anxiety. *Expert Rev Respir Med.* (2021) 15:5–7. 10.1080/17476348.2020.1787835 32578464

[35] Huang Y Zhao N. Generalized anxiety disorder, depressive symptoms and sleep quality during Covid-19 outbreak in China: a web-based cross-sectional survey. *Psychiatry Res.* (2020) 288:112954. 10.1016/j.psychres.2020.112954 32325383 PMC7152913

[36] Ovsyannikov ES Avdeev SN Budnevsky AV Shkatova YS. Influence of anxiety/depression on the subjective evaluation of cough in patients with chronic obstructive pulmonary disease and obesity. *Medicina (Kaunas).* (2019) 55:134. 10.3390/medicina55050134 31091811 PMC6572558

[37] Stage KB Middelboe T Pisinger C. Depression and chronic obstructive pulmonary disease (Copd). Impact on survival. *Acta Psychiatr Scand.* (2005) 111:320–3. 10.1111/j.1600-0447.2004.00497.x 15740469

[38] Ng TP Niti M Tan WC Cao Z Ong KC Eng P. Depressive symptoms and chronic obstructive pulmonary disease: effect on mortality, hospital readmission, symptom burden, functional status, and quality of life. *Arch Intern Med.* (2007) 167:60–7. 10.1001/archinte.167.1.60 17210879

[39] Barnes PJ Celli BR. Systemic manifestations and comorbidities of Copd. *Eur Respir J.* (2009) 33:1165–85. 10.1183/09031936.00128008 19407051

[40] Herbert TB Cohen S. Depression and immunity: a meta-analytic review. *Psychol Bull.* (1993) 113:472–86. 10.1037/0033-2909.113.3.472 8316610

[41] Bailey PH. The dyspnea-anxiety-dyspnea cycle–copd patients’ stories of breathlessness: “it’s scary/when you can’t breathe”. *Qual Health Res.* (2004) 14:760–78. 10.1177/1049732304265973 15200799

[42] Ciechanowski PS Katon WJ Russo JE. Depression and diabetes: impact of depressive symptoms on adherence, function, and costs. *Arch Intern Med.* (2000) 160:3278–85. 10.1001/archinte.160.21.3278 11088090

[43] Moise N Ye S Alcantara C Davidson KW Kronish I. Depressive symptoms and decision-making preferences in patients with comorbid illnesses. *J Psychosom Res.* (2017) 92:63–6. 10.1016/j.jpsychores.2015.12.001 26682488 PMC4889561

[44] Lin FL Yeh ML Lai YH Lin KC Yu CJ Chang JS. Two-month breathing-based walking improves anxiety, depression, dyspnoea and quality of life in chronic obstructive pulmonary disease: a randomised controlled study. *J Clin Nurs.* (2019) 28:3632–40. 10.1111/jocn.14960 31192478

[45] Li Z Liu S Wang L Smith L. Mind-body exercise for anxiety and depression in copd patients: a systematic review and meta-analysis. *Int J Environ Res Public Health.* (2019) 17:22. 10.3390/ijerph17010022 31861418 PMC6981896

[46] Gordon CS Waller JW Cook RM Cavalera SL Lim WT Osadnik CR. Effect of pulmonary rehabilitation on symptoms of anxiety and depression in Copd: a systematic review and meta-analysis. *Chest.* (2019) 156:80–91. 10.1016/j.chest.2019.04.009 31034818

[47] Kuint R Cohen Goichman P Mizrachi A Breuer R Abutbul A Berkman N The effect of a multidisciplinary integrated approach on outcomes in chronic obstructive pulmonary disease. *Israel Med Assoc J.* (2020) 22:761–4. 33381948

