# Supplementary material for: Temporal trends in anxiety and depression prevalence and their association with adverse outcomes in patients hospitalized for acute exacerbations of chronic obstructive pulmonary disease in Beijing, China, from 2004 to 2020
Source: Front Psychiatry. 2022 Oct 31;13:996451. doi: 10.3389/fpsyt.2022.996451 (PMC9659583; doi:10.3389/fpsyt.2022.996451)
Supplement: Supplementary file 1 [file Data_Sheet_1.docx]

Supplementary Material

# Supplementary Tables

**Supplementary Table 1. The types of anxiety and depression diagnosis and the corresponding ICD-10 code of patients hospitalized for AECOPD from 2004 to 2020 in Beijing, China.**

|  | Diagnosis | ICD-10 code | Number |
| --- | --- | --- | --- |
| Depression | Mild depressive episode | F32.0 | 27 |
|  | Moderate depressive episode | F32.1 | 3 |
|  | Depressive episode, unspecified | F32.9 | 3450 |
|  | Recurrent depressive disorder, current episode moderate | F33.1 | 1 |
|  | Recurrent depressive disorder, unspecified | F33.9 | 3 |
|  | Mixed anxiety and depressive disorder | F41.2 | 1034 |
| Anxiety | Specific (isolated) phobias | F40.2 | 7 |
|  | Panic disorder [episodic paroxysmal anxiety] | F41.0 | 18 |
|  | Generalized anxiety disorder | F41.1 | 3269 |
|  | Mixed anxiety and depressive disorder | F41.2 | 1034 |
|  | Other mixed anxiety disorders | F41.3 | 5 |
|  | Anxiety disorder, unspecified | F41.9 | 285 |

ICD-10, International Classification of Diseases, 10th version.

**Supplementary Table 2. The characteristics and the overall prevalence of only depression, only anxiety, both of depression and anxiety among patients hospitalized for AECOPD from 2004 to 2020 in Beijing, China.**

|  | Total | Only depression | |  | Only anxiety | |  | Both depression and anxiety | |
| --- | --- | --- | --- | --- | --- | --- | --- | --- | --- |
|  |  | N | *P* |  | N | *P* |  | N | *P* |
| Overall | 382125 | 2911 (0.8%) |  |  | 3288 (0.9%) |  |  | 1713 (0.4%) |  |
| Gender |  |  |  |  |  |  |  |  |  |
| Male | 252055 | 1721 (0.7%) | <0.001 |  | 1773 (0.7%) | <0.001 |  | 944 (0.4%) | <0.001 |
| Female | 130070 | 1190 (0.9%) |  |  | 1515 (1.2%) |  |  | 769 (0.6%) |  |
| Age group |  |  |  |  |  |  |  |  |  |
| 20-59 years | 28893 | 153 (0.5%) | <0.001 |  | 137 (0.5%) | <0.001 |  | 77 (0.3%) | <0.001 |
| 60-74 years | 125190 | 770 (0.6%) |  |  | 900 (0.7%) |  |  | 475 (0.4%) |  |
| ≥75 years | 228042 | 1988 (0.9%) |  |  | 2251 (1.0%) |  |  | 1161 (0.5%) |  |
| P trend |  |  | <0.001 |  |  | <0.001 |  |  | <0.001 |
| Institute level | | | | | | | | | |
| Secondary hospitals | 108861 | 1050 (1.0%) | <0.001 |  | 903 (0.8%) | 0.223 |  | 471 (0.4%) | 0.549 |
| Tertiary hospitals | 269824 | 1815 (0.7%) |  |  | 2347 (0.9%) |  |  | 1206 (0.4%) |  |
| Charlson Comorbidity Index | | | | | | | | | |
| 0 | 148424 | 1191 (0.8%) | 0.010 |  | 1370 (0.9%) | 0.004 |  | 621 (0.4%) | 0.029 |
| 1 | 128378 | 987 (0.8%) |  |  | 1045 (0.8%) |  |  | 624 (0.5%) |  |
| ≥2 | 105157 | 733 (0.7%) |  |  | 873 (0.8%) |  |  | 468 (0.4%) |  |
| P trend |  |  | 0.003 |  |  | 0.007 |  |  | 0.022 |

Institute level missing (n=3440), Charlson Comorbidity Index missing (n=166).

**Supplementary Table 3. The prevalence of anxiety and/or depression among patients hospitalized for AECOPD from 2004 to 2020 in Beijing, China.**

| Year | Total | With depression and/or anxiety | Subtypes | | |
| --- | --- | --- | --- | --- | --- |
|  |  |  | Only depression | Only anxiety | Both |
| 2004 | 4096 | 11 (0.3%) | 2 (0.0%) | 9 (0.2%) | 0 (0.0%) |
| 2005 | 7024 | 27 (0.4%) | 7 (0.1%) | 19 (0.3%) | 1 (0.0%) |
| 2006 | 9229 | 54 (0.6%) | 16 (0.2%) | 33 (0.4%) | 5 (0.0%) |
| 2007 | 12913 | 77 (0.6%) | 19 (0.2%) | 56 (0.4%) | 2 (0.0%) |
| 2008 | 16440 | 95 (0.6%) | 5 (0.0%) | 84 (0.5%) | 6 (0.0%) |
| 2009 | 19289 | 120 (0.6%) | 8 (0.0%) | 100 (0.5%) | 12 (0.1%) |
| 2010 | 21977 | 187 (0.9%) | 15 (0.1%) | 141 (0.6%) | 31 (0.1%) |
| 2011 | 22701 | 332 (1.5%) | 60 (0.3%) | 205 (0.9%) | 67 (0.3%) |
| 2012 | 30868 | 757 (2.5%) | 281 (0.9%) | 371 (1.2%) | 105 (0.3%) |
| 2013 | 36381 | 918 (2.5%) | 382 (1.1%) | 350 (1.0%) | 186 (0.5%) |
| 2014 | 41605 | 999 (2.4%) | 413 (1.0%) | 383 (0.9%) | 203 (0.5%) |
| 2015 | 28163 | 782 (2.8%) | 338 (1.2%) | 257 (0.9%) | 186 (0.5%) |
| 2016 | 30466 | 869 (2.9%) | 383 (1.3%) | 260 (0.9%) | 226 (0.7%) |
| 2017 | 30604 | 752 (2.5%) | 311 (1.0%) | 255 (0.8%) | 186 (0.6%) |
| 2018 | 27804 | 718 (2.6%) | 256 (0.9%) | 302 (1.1%) | 160 (0.6%) |
| 2019 | 27755 | 758 (2.7%) | 262 (0.9%) | 292 (1.1%) | 204 (0.7%) |
| 2020 | 14810 | 456 (3.1%) | 153 (1.0%) | 171 (1.2%) | 132 (0.9%) |

**Supplementary Table 4. The multivariate analyses of the associations between only depression, only anxiety, both anxiety and depression on in-hospital outcomes and the risks of readmission for AECOPD after discharge.**

|  | Model 1 | | |  | Model 2 | | |
| --- | --- | --- | --- | --- | --- | --- | --- |
|  | odds ratio (95% CI) | *P* | *P* for difference among subtypes |  | odds ratio (95% CI) | *P* | *P* for difference among subtypes |
| Receiving mechanical ventilation ^a^ |  |  |  |  |  |  |  |
| Without depression nor anxiety | reference |  |  |  | reference |  |  |
| Only depression | 0.99 (0.78, 1.27) | 0.953 | 0.181 |  | 1.07 (0.83, 1.36) | 0.608 | 0.090 |
| Only anxiety | 1.33 (0.65, 2.70) | 0.435 |  |  | 1.31 (0.64, 2.71) | 0.460 |  |
| Both | 0.72 (0.52, 1.00) | 0.050 |  |  | 0.73 (0.53, 1.02) | 0.064 |  |
| In-hospital mortality ^b^ |  |  |  |  |  |  |  |
| Without depression nor anxiety | reference |  |  |  | reference |  |  |
| Only depression | 0.69 (0.52, 0.90) | 0.007 | 0.437 |  | 0.74 (0.56, 0.98) | 0.033 | 0.531 |
| Only anxiety | 0.81 (0.64, 1.03) | 0.089 |  |  | 0.80 (0.62, 1.01) | 0.065 |  |
| Both | 0.63 (0.45, 0.90) | 0.012 |  |  | 0.64 (0.45, 0.92) | 0.015 |  |
| Length of hospital stay |  |  |  |  |  |  |  |
| Without depression nor anxiety | reference |  |  |  | reference |  |  |
| Only depression | 1.07 (1.01, 1.13) | 0.016 | 0.066 |  | 1.08 (1.03, 1.13) | 0.004 | 0.020 |
| Only anxiety | 1.19 (1.12, 1.27) | <0.001 |  |  | 1.20 (1.13, 1.27) | <0.001 |  |
| Both | 1.09 (1.03, 1.16) | 0.007 |  |  | 1.10 (1.04, 1.18) | 0.003 |  |
| Medical cost |  |  |  |  |  |  |  |
| Without depression nor anxiety | reference |  |  |  | reference |  |  |
| Only depression | 1.04 (0.98, 1.11) | 0.159 | 0.171 |  | 1.10 (1.04, 1.16) | <0.001 | 0.371 |
| Only anxiety | 1.17 (1.08, 1.27) | <0.001 |  |  | 1.17 (1.09, 1.25) | <0.001 |  |
| Both | 1.05 (0.99, 1.12) | 0.077 |  |  | 1.08 (1.02, 1.14) | 0.008 |  |
| 30-day readmission for AECOPD ^c^ |  |  |  |  |  |  |  |
| Without depression nor anxiety | reference |  |  |  | reference |  |  |
| Only depression | 1.15 (0.87, 1.53) | 0.317 | 0.540 |  | 1.24 (0.92, 1.66) | 0.164 | 0.456 |
| Only anxiety | 1.06 (0.88, 1.28) | 0.559 |  |  | 1.29 (1.00, 1.43) | 0.056 |  |
| Both | 1.12 (0.79, 1.58) | 0.535 |  |  | 1.25 (0.90, 1.75) | 1.880 |  |
| 90-day readmission for AECOPD ^c^ |  |  |  |  |  |  |  |
| Without depression nor anxiety | reference |  |  |  | reference |  |  |
| Only depression | 1.28 (1.05, 1.57) | 0.014 | 0.347 |  | 1.36 (1.10, 1.68) | 0.004 | 0.256 |
| Only anxiety | 1.22 (1.07, 1.39) | 0.003 |  |  | 1.35 (1.19, 1.53) | <0.001 |  |
| Both | 1.39 (1.09, 1.75) | 0.007 |  |  | 1.52 (1.22, 1.90) | <0.001 |  |
| 180-day readmission for AECOPD ^c^ |  |  |  |  |  |  |  |
| Without depression nor anxiety | reference |  |  |  | reference |  |  |
| Only depression | 1.39 (1.18, 1.63) | <0.001 | 0.090 |  | 1.45 (1.22, 1.72) | <0.001 | 0.070 |
| Only anxiety | 1.24 (1.11, 1.40) | <0.001 |  |  | 1.35 (1.20, 1.52) | <0.001 |  |
| Both | 1.49 (1.21, 1.82) | <0.001 |  |  | 1.61 (1.33, 1.95) | <0.001 |  |
| 1-year readmission for AECOPD ^c^ |  |  |  |  |  |  |  |
| Without depression nor anxiety | reference |  |  |  | reference |  |  |
| Only depression | 1.42 (1.24, 1.64) | <0.001 | 0.146 |  | 1.48 (1.28, 0.71) | <0.001 | 0.087 |
| Only anxiety | 1.36 (1.20, 1.54) | <0.001 |  |  | 1.45 (1.28, 1.63) | <0.001 |  |
| Both | 1.59 (1.33, 1.91) | <0.001 |  |  | 1.70 (1.43, 2.01) | <0.001 |  |

Model 1: adjusted for admission year;

Model 2: adjusted for sex, continuous age, continuous Charlson Comorbidity Index, institute level and admission year.

CI, confidence interval; ^a^, hospitalization recodes in 2012-2020, N=26,8456; ^b^, hospitalization recodes in 2007-2020, N=36,1776; ^c^, hospitalization recodes alive at discharge in 2004 to 2019, N=35,6862.

# Supplementary Figures

**Supplementary Figure 1. The prevalence of only anxiety among patients hospitalized for AECOPD in Beijing, stratified by gender, age group, and institute level. Caption: (A) gender; (B) age group; (C) institute level.**

**Supplementary Figure 2. The prevalence of only depression among patients hospitalized for AECOPD in Beijing, stratified by gender, age group, and institute level. Caption: (A) gender; (B) age group; (C) institute level.**

**Supplementary Figure 3. The prevalence of both anxiety and depression among patients hospitalized for AECOPD in Beijing, stratified by gender, age group, and institute level. Caption: (A) gender; (B) age group; (C) institute level.**
